# Prototype analysis of a low-power, small-scale wearable medical device

**DOI:** 10.2478/joeb-2024-0020

**Published:** 2025-01-04

**Authors:** Pablo Dutra da Silva, Pedro Bertemes Filho

**Affiliations:** 1 Electrical Engineering Department, State University of Santa Catarina, Santa Catarina, Brazil

**Keywords:** Wearable, glucose noninvasive measuring, enhanced Howland current source, low voltage power supply, instrumentation amplifier

## Abstract

Wearable and portable devices are gaining significant popularity across consumer electronics as well as in medical and industrial fields. To ensure that these devices are both comfortable and appealing to users, they need to have low battery consumption and be compact in both size and weight. The EGluco project is focused on developing a wearable device for non-invasive blood glucose monitoring. This multi-sensor device incorporates electrical bioimpedance spectroscopy as one of its measurement techniques. One of the earlier versions of the device was deemed unsuitable as a wearable due to its large size and high power consumption. To make the device more suitable for wearability, the previous hardware was assessed, and a new design was proposed that simplified the system’s power supply and reduced the operating voltage. This article presents two of these designs: an improved Howland current source with a supply voltage of 3.3 V, an output current of 250 *μA*, and the ability to conduct bioimpedance analysis up to 1 MHz using pulsed DIBS (Discrete Interval Binary Sequence) signals, and an instrumentation amplifier with the same supply voltage as the current source, a voltage gain of four, and a slew rate of 150 *V/μs*. By simplifying the power supply and implementing other changes, the device’s size was reduced to a single 5 × 5 cm circuit board, compared to the previous configuration of four separate boards connected by cables.

## Introduction

The wearable device is rapidly becoming a crucial tool in health monitoring, medical diagnostics, and selfquantification, especially for athletes and health enthusiasts. This type of device refers to anything worn by an individual that has the capability to measure biological signals and activity patterns. Typically designed as wristbands, smartwatches, chest straps, shoes, socks, or glasses, wearables have transitioned from primarily tracking activities such as sleep, walking, and running to functioning as medical instruments capable of measuring various vital signs. Early versions of wearable devices were not suitable for medical diagnostics; however, recent scientific research has begun exploring their potential to detect correlations between activity patterns—linked to life-style—and specific cardiovascular diseases. The goal for wearable technology is to provide non-invasive, real-time monitoring for patients at home or in ambulatory settings, offer early alerts or diagnoses for healthy individuals, and enhance performance for athletes [1, 2].

Recent technological advancements have expanded the capabilities of wearables by incorporating additional sensors that measure biological signals such as heart rate, blood oxygen levels, electrocardiograms, and skin temperature. Some of these devices are commercially available, including those from companies like Apple, Samsung, Xiaomi, Fitbit, and Garmin, while others are still in the research and development phase [1, 2]. A significant area of research involves utilizing these commercial wearable models for diagnosing cardiovascular problems [1, 2]. However, for biological parameters like glucose, there is no non-invasive commercial solution that matches the performance of the gold standard, which is an invasive electrochemical method [3, 4, 5]. This gap in technology supports the ongoing scientific efforts to develop non-invasive glucose measurement systems [3, 4, 5, 6]. The minimally invasive CGM (Continuous Glucose Monitor) from Abbott Labs (FreeStyle Libre) has improved diabetes management but differs from wearables in its function, as it lacks the multi-functional capabilities of a wearable device. Furthermore, CGM requires frequent electrode replacements, making it costly for patients, unlike non-invasive electrodes, which do not require such replacements.

Diabetes Mellitus is a chronic condition characterized by inadequate insulin production or absorption, a hormone responsible for regulating blood glucose by breaking down glucose molecules and providing energy to the body [7]. Managing diabetes involves constant monitoring of blood glucose levels, enabling patients to take effective actions to control their glucose levels and mitigate the disease’s complications [4, 5, 6]. Currently, electrochemical sensors used in glucose meters require blood samples, which must be placed on reagent strips that are then inserted into an electronic device to determine glucose concentration. While this method remains the gold standard for selfmonitoring [4, 5, 6], it is painful and unhygienic, requiring frequent finger pricks to obtain blood. Additionally, diabetes patients must perform this procedure multiple times a day.

Consequently, a non-invasive glucose monitor would offer significant benefits for diabetes patients, including pain reduction, infection prevention, and cost savings, especially if the monitor is designed as a wearable device that provides automatic measurements with accuracy comparable to the gold standard [8, 9].

A wearable glucose measurement system should, like other wearables, feature ultra-low voltage supply, minimal power consumption, compact size, and high accuracy [10, 11, 12, 13]. These characteristics are typically achieved through very large-scale integration (VLSI) technologies such as complementary metal-oxide semiconductor (CMOS), which is a standard method for fabricating integrated circuits (ICs). Several research papers propose different IC designs for biomedical applications, including those for glucose monitoring [8, 12]. One major challenge in wearables is power consumption, which impacts battery usage. As a result, researchers have been investigating energy harvesting techniques to power medical wearables more efficiently [10].

To advance the development of non-invasive, wearable blood glucose monitoring for diabetics, this work continues the research from [14, 15, 16], focusing on an IC design powered by batteries. This research direction is essential since Teixeira’s [16] earlier work used discrete components on a prototype printed circuit board (PCB) to validate the EGluco project [17]. The initial prototype consists of two connected boxes: the first holds the sensors, bioimpedance electrodes, and signal conditioning circuits, while the second contains an STM32 dev kit, a Bluetooth module, a 9V battery, and a medical power supply to deliver ±5*V* to the first box and +5*V* to the second box. The goal is to redesign this system to run off 3.3 *V* batteries as the sole power source, using an Enhanced Howland Current Source (EHCS) and a instrumentation amplifier as the analog blocks for bioimpedance analysis. Moreover, the system’s volume has been reduced by consolidating everything onto a single 5 × 5 × 2 cm PCB, utilizing a microcontroller and Bluetooth module compatible with the 3.3 *V* supply.

This paper presents the design considerations and outcomes of a low-power, single-supply Enhanced Howland Current Source and an instrumentation amplifier for a wearable glucose monitor using bioimpedance spectroscopy as one of the analysis techniques. Section 2 discusses the current prototype, while Section 3 introduces the EHCS for low-voltage power supplies and the instrumentation amplifier. Finally, Section 4 presents the test results for both circuits presented.

## Noninvasive Bioimpedance Glucometer: current prototype

A non-invasive blood glucose meter based on bioimpedance spectroscopy has been developed as part of the EGluco project [17]. Several aspects of this development are discussed in the works [14, 15, 16]. Analytical models for determining blood glucose levels from photoplethysmography analysis are outlined in [15], while models for electrical bioimpedance analysis are provided in [14]. Despite the significance of these analytical models, current development is shifting towards the application of artificial intelligence (AI) for glucose level estimation. Although the first prototype was not suitable as a wearable device, it provides essential data for training AI algorithms. Furthermore, the initial prototype is crucial for defining system specifications, assessing the sensitivity of glucose measurements to factors such as skin temperature, humidity, heart rate, and oxygen saturation, and verifying the connections between different parts of the system.

**Figure 1:**
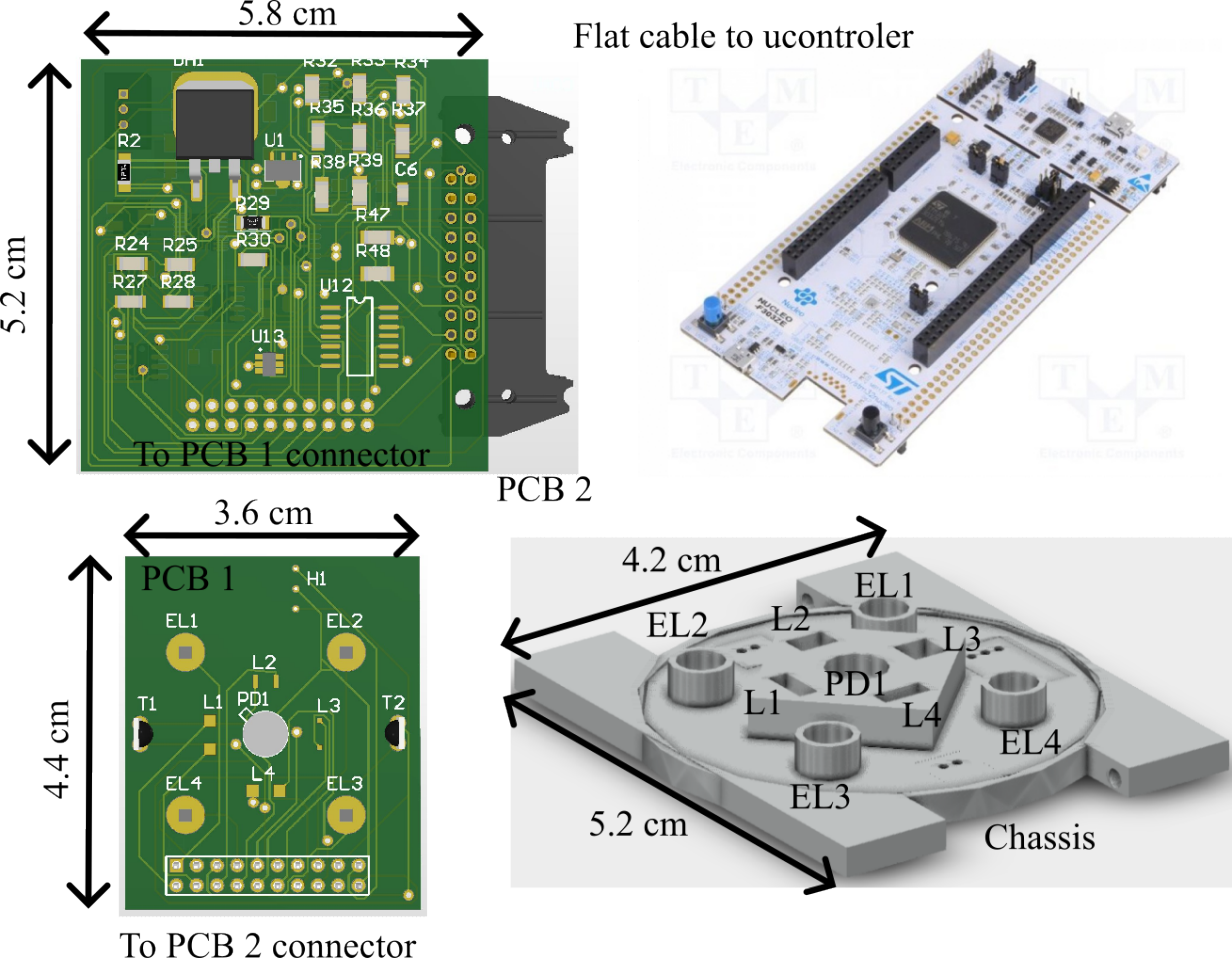
System diagram for the current device under development.

The work presented in [16] details a system based on printed circuit board (PCB) technology, utilizing commercial integrated circuits (ICs) and discrete passive components. In Figure 1, some components of the system are visible, including two PCBs (PCB1 and PCB2), a chassis for sensor positioning, and the STM32 evaluation board. A DC ±5 *V* power supply unit and a Bluetooth module, which are not shown in Figure 1, complete the system. To facilitate bioimpedance analysis, the terminals (EL1 – EL4) on PCB1 must pass through corresponding apertures (EL1 – EL4) in the chassis to make contact with the patient’s skin. The connection between PCB1 and PCB2 is established through a pin header connector, as shown in Figure 1. These two PCBs and the chassis form a wristband, which is connected to an external microcontroller evaluation board via a flat cable attached to PCB2. Additionally, a Bluetooth module is directly connected to the microcontroller evaluation board via a pin header. This prototype represents only a portion of the full system, and it cannot be considered a complete wearable device. Moreover, the physical dimensions of the PCBs are larger than any commercial wearable device. Thus, a system analysis should be conducted to identify the modifications necessary to reduce the size and make the device suitable for wearability.

**Figure 2:**
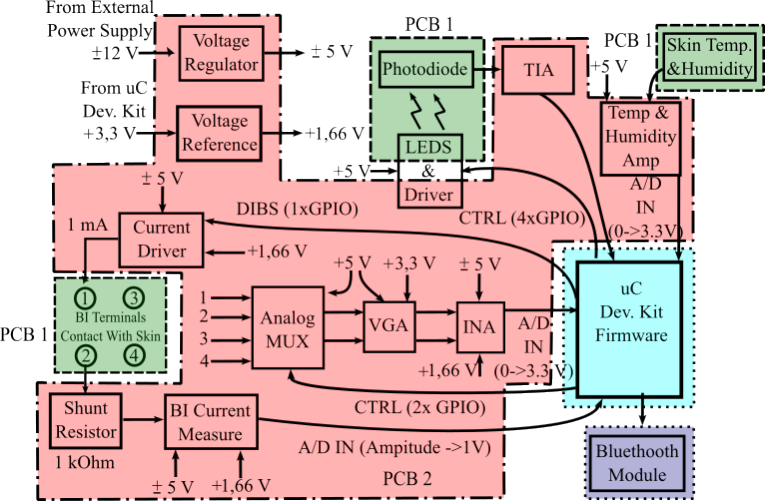
System diagram for the current device under development.

This system analysis can be conducted by referring to Figure 2, which organizes the system blocks similarly to the physical layout shown in Figure 1. PCB1 (indicated by green with dashed borders) is designed to serve as the soldering base for the sensors and actuators, connecting the analog signal conditioning circuitry and control signals passing through PCB2. For bioimpedance analysis, four terminals (EL1, EL2, EL3, and EL4) are identified in Figure 1 and numbered 1 through 4 in Figure 2. Terminals 1 and 2 are used to inject current into the user’s skin. Terminal 2 connects to a shunt resistor for current measurement. Terminals 3 and 4 are used to measure the voltage at a position displaced from the current injection points, which is necessary for evaluating the skin impedance. Additionally, four LEDs and a photodiode for the photoplethysmography process are attached to PCB1, along with temperature and humidity sensors. These sensors are used to measure oxygen saturation, skin temperature, and humidity, which help detect the measurement context. This contextual information is crucial for understanding skin conditions and other variables that could impact glucose measurement via bioimpedance analysis. All of these sensors and actuators are connected to the analog conditioning circuitry on PCB2 via a pin header.

Analog signal conditioning and power supply management circuitry are housed on PCB2 (indicated by the red dashed border). This PCB is also responsible for controlling signals between the microcontroller and the sensors and other circuits. A voltage regulator on PCB2 converts an external ±12*V* supply to a ±5*V* power supply for the analog circuits. Additionally, the power management unit uses the +3 *V* supply from the microcontroller evaluation kit to generate a +1.6 *V* reference voltage for certain analog circuits. This reference voltage is used to offset signals being conditioned for the microcontroller’s analog-to-digital converter (ADC). The analog signal conditioning circuits, which are primarily for bioimpedance analysis, include an analog multiplexer, a variable gain amplifier (which is a future addition and is not part of the current prototype), and an instrumentation amplifier to measure the voltage between terminals 1-2 and 3-4. The bioimpedance analyzer also contains a current driver and a current measuring circuit with a shunt resistor and voltage amplifier. The optical signal conditioning system includes a trans-impedance amplifier (TIA), and the temperature and humidity measurement circuits use voltage amplifiers.

The outputs of these amplifiers are connected to the STM32 evaluation kit (represented in light blue with dotted borders), where the firmware processes all of the signals. This evaluation board requires a 3.3 *V* power supply and has two communication interfaces: one via a USB for connecting to a computer and another through Bluetooth for sending data to smartphones. Figure 2 illustrates the various voltage levels distributed across the PCB, resulting in a more complex power management system. This complexity in power management creates challenges for battery-powered systems, such as wearables, due to volume limitations and energy inefficiencies. Moreover, the growing number of variables to be measured increases the number of chips required for signal conditioning. As a result, the use of multiple boards increases both the cost and energy consumption of the system.

## Design of a Enhanced Howland Current Source and a instrumentation amplifier for battery low voltage power supply

The bioimpedance spectroscopy system must meet the following criteria: a single power supply of +3.3 *V*; an output current around 250 *μAp*; an output impedance greater than 10 M© and a single input pulsed signal (DIBS) with a maximum amplitude of +3.3 *V* for the current source. A single power supply of +3.3 *V*, a Slew Rate of 150 *V*/*μ*s a voltage gain of four are the specifications for the instrumentation amplifier.

**Figure 3:**
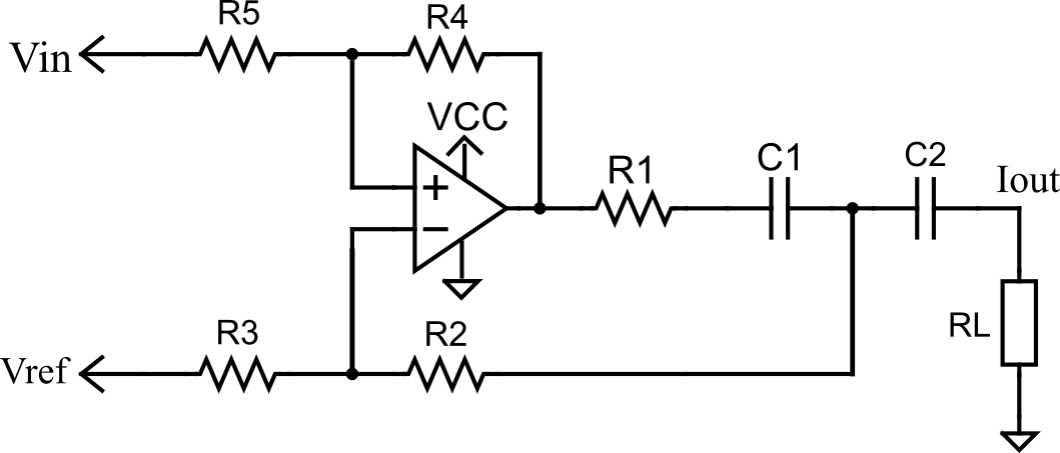
Enhanced Howland Current source Schematic.

The Enhanced Howland Current Source (EHCS) is shown in Fig. 3. This configuration is commonly used in both time and frequency domain impedance spectroscopy for injecting current into the sample [18, 19, 20, 21, 22]. When the resistors are properly matched, the EHCS is known for its high output impedance. As a result, resistor tolerances of less than 0.1% are necessary when using discrete resistors [23, 24, 25].

In both frequency and time domain spectrographic analysis, the EHCS is suitable for generating sinusoidal and pseudo-random current signals. It is important to carefully evaluate the operational amplifier’s gainbandwidth product and slew rate when used in timedomain BIS. The design equations for the EHCS are well-documented in the impedance spectroscopy literature and are not repeated here. The output current, as defined by equation (1), depends on the input differential voltage and resistor R1 when *R*4 = *R*5 and *R*3 = *R*2+*R*1, as depicted in Fig. 3 [23, 24, 25]. Additionally, the accuracy of the input voltage source is critical because of the EHCS’s role as a voltage-to-current converter.

1 $$lout=\frac{\;Vin-\;Vref}{R1}$$

The OPA2354 operational amplifier provides a gainbandwidth product of 250 *MHz* and a slew rate of 150 *V*/*μ*s. It operates in a rail-to-rail configuration at the output and requires a minimum single supply voltage of 2.5 *V*. These specifications are ideal for processing DIBS signals at frequencies up to 1 *MHz*. This amplifier consumes a quiescent current of 4.9 *mA*, which is about twenty times the specified output current, and this current is drawn continuously when the circuit is powered on. The output current design of the EHCS takes into account the input signal voltage levels (Vin_high_ = *V CC* and *V ref* = *V CC*/2) and their relationship with resistor R1, as described in equation (1). This results in a resistor value of 6.6 *K*Ω. The final design includes resistors *R*4 = *R*5 = 10 *K*Ω, *R*2 = 100 Ω, and *R*3 = 6.7 *K*Ω, along with DC decoupling capacitors C1 = C2 = 20 *μ*F.

**Figure 4:**
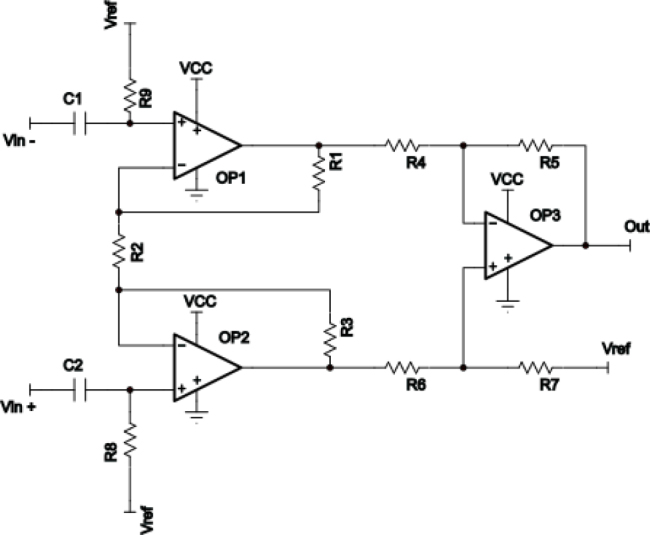
Instrumentation Amplifier Schematic.

The schematic diagram of the instrumentation amplifier is shown in Fig. 4. It can be observed that a topology using three operational amplifiers was chosen. This decision was made because it was difficult to find monolithic instrumentation amplifiers with low supply voltages that had a slew rate compatible with the application available on the market. For a supply voltage of 3.3V, only operational amplifiers were found. Therefore, the topo-logy using three operational amplifiers from the OPA4354 chip, which has the same electrical characteristics as the OPA2354 used for the improved Howland current source, was selected.

This topology is necessary because the input impedance of the instrumentation amplifier is higher than that of a difference amplifier with only one operational amplifier. The resistor values chosen are: R1 to R7 = 10 KΩ, R8 and R9 = 10 MΩ, and the decoupling capacitors C1 and C2 = 10 *μ*F. As shown in Fig. 4, the gain of this instrumentation amplifier is four, and the inputs are biased with the voltage Vref so that the output signal has an average value of VCC/2.

### Ethical approval

The conducted research is not related to either human or animal use.

## Results and discussions

**Figure 5:**
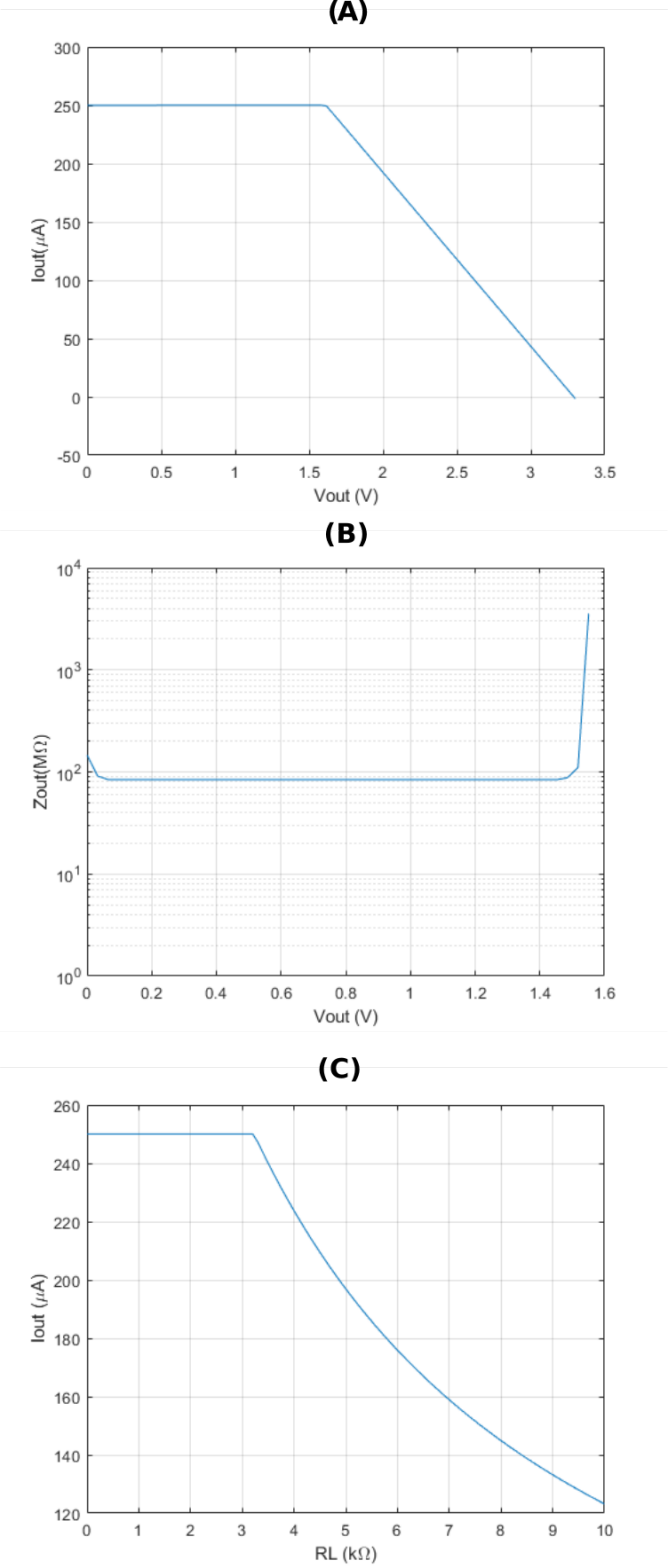
DC simulation results for the low power supply design.

The data shown in Fig. 5 (A), Fig. 5 (B), and Fig. 5 (C) were obtained through a Cadence PSPICE simulation using the op-amp macromodel supplied by the manufacturer, specifically for the EHCS scenario. The correlation between the output DC current and the output DC voltage sweep is derived when a voltage source is connected to the load electrodes, as illustrated in Fig. 5 (A) and Fig. 5 (B). These experiments demonstrate the allowable output voltage swing. Fig. 5 (A) and Fig. 5 (B) indicate that the EHCS allows a swing of +1.6 V for a power supply voltage of +3.3 *V*, and the output DC impedance reaches approximately ≈ 200 MΩ. Fig. 5 (C) reveals that the maximum load resistance is about ≈ 3 *K*Ω. The DC sweep analysis confirms the output voltage swing and the load limits necessary to maintain the desired output current.

Although the EHCS achieved a DC output impedance of approximately 200 *M*Ω within a specific DC operating range, this value depends on the resistor tolerance and the op-amp’s second-order effects. To assess how the EHCS responds to temperature changes and voltage supply variations, minimalistic corner simulations were conducted. Table 1 summarizes the verification of the DC output current across the specified temperature range of 0 to 100°C and voltage supply range of 1.8 to 3.3*V*. Results show that there is no significant change in the current over the analyzed temperature and supply voltage ranges. The relationship between the resistor tolerance and the output of the current source was evaluated through a Monte Carlo simulation. In the PSPICE simulations, using the op-amp macromodel provided by the manufacturer, the output current varied from 246 to 267 *μ*A for a 1% resistor tolerance, and from 249.7 to 250.55*μ*A for a 0.1% tolerance. The latter value was used for the prototype.

**Table 1:** CC output current (in *μ*A) considering temperature and voltage supply corners.

Topology	Iout (0<*T*<100°*C*)	Iout (1.8<*VCC*<3.3*V*)
EHCS	250.118 to 250.121	249.97 to 250.12

**Figure 6:**
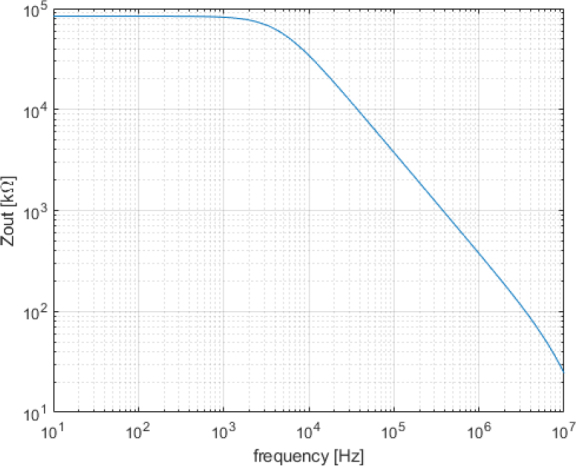
Output impedance versus frequency in AC analysis.

The results from the AC analysis of the output impedance are presented in Fig. 6. This analysis was conducted using the same circuit configuration as the DC analysis, with the load replaced by a signal generator in SPICE for an AC sweep analysis. The EHCS performs well from DC to 1 *MHz*, where the output impedance remains above 300 *K*Ω, which is still related to the op-amp’s performance. Moreover, this value is about one hundred times higher than the maximum load resistance of ≈ 3*K*Ω, which results in only a minor error due to the output impedance. It’s important to note that resistor tolerances can impact the EHCS output impedance, leading to degradation.

**Figure 7:**
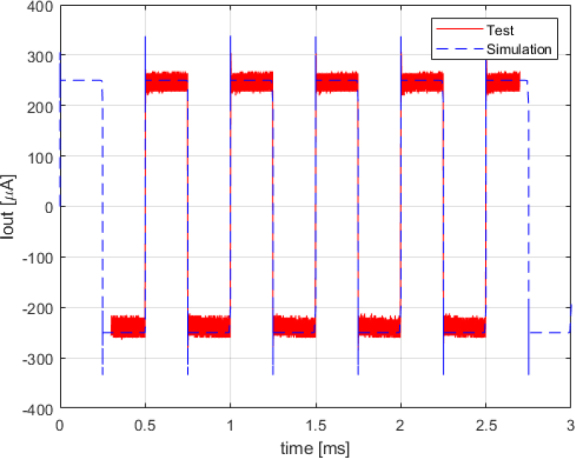
Simulation vs test output current comparison for 2 : *kHz* rectangle wave.

The tests were performed with a load of 1 *K*Ω using rectangular signals. This choice was made because the system was designed and optimized for Discrete Interval Binary Sequence (DIBS) signals, which exhibit pulsed behavior. Fig. 7 compares the bench test results with the PSPICE simulation for an input signal of 2 *kHz* and a voltage level of 3.3 *V*. The comparison shows that despite some amplitude noise, the experimental results closely match the simulation, demonstrating the accuracy of the current source design.

**Figure 8:**
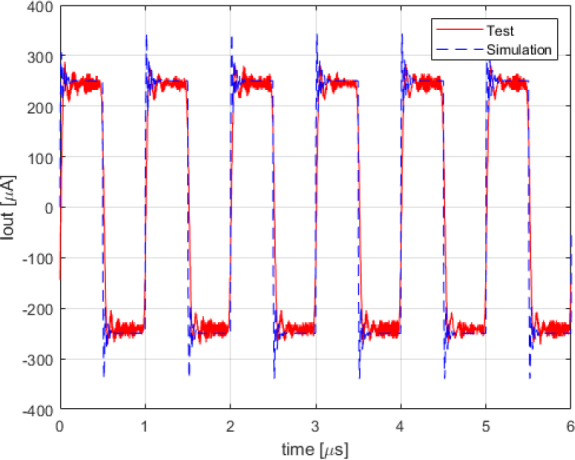
Simulation vs test output current comparison for 1 *MHz* rectangle wave.

As observed in Fig. 6, even at 1 *MHz* (as shown in Fig. 8), the modulus of the output impedance does not substantially affect the accuracy of the output current, which remains at approximately 250 *μ*A.

**Figure 9:**
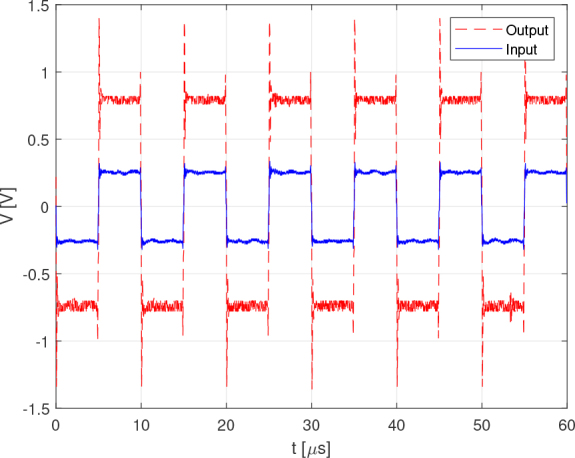
Instrumentation amplifier test with a 1 *K*Ω impedance for a 100 *kHz* rectangle wave.

For the tests with the instrumentation amplifier, a load of 1 *K*Ω was first used. This was connected to the current source and the input of the instrumentation amplifier with a bipolar electrode configuration. The signal used for the tests was a rectangular digital signal with a maximum voltage (high level) of 3.3 *V*. Fig. 9 shows the results for this test, comparing the voltage at the load resistor (Input) and the voltage at the output of the instrumentation amplifier (Output). For comparison purposes, the average value (*VCC*/2) was taken from the instrumentation amplifier’s output voltage signal to make it easier to compare the signals. It can be concluded that, for a frequency of 100 kHz, the amplifier functions correctly, showing linearity and the designed gain.

**Figure 10:**
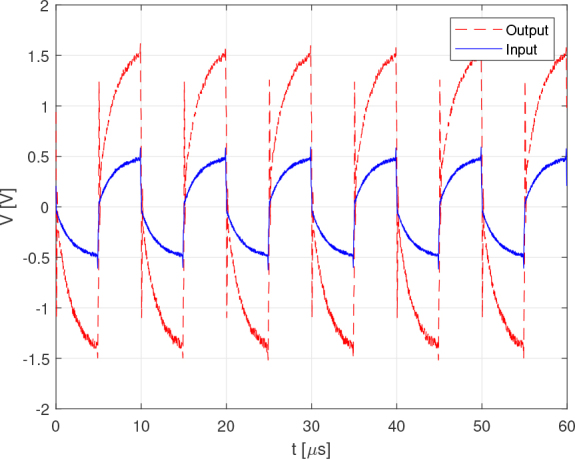
Instrumentation amplifier test with a Cole impedance for a 100 *kHz* rectangle wave.

A test using an equivalent Cole model with two 1 kΩ resistors and a 1 nF capacitor was carried out and the results are shown in Fig. 10. All the conditions were maintained with respect to the test shown in Fig. 4 except the load. Fig. 10 shows that the amplifier maintains its linearity and the gain designed for the Cole load up to a frequency of 100 kHz. Both the test whose results are shown in Fig. 4 and the test whose results are shown in Fig. 10 demonstrate that the analog front end for electrical impedance spectroscopy is capable of taking accurate measurements up to a frequency of 100kHZ.

The tests carried out with frequencies below 100 kHz maintained the same conclusions as those presented for Fig. 4 and Fig. 10. For frequencies up to 1 *MHz*, the error may increase due to the degree of distortion shown in the results for both loads.

**Figure 11:**
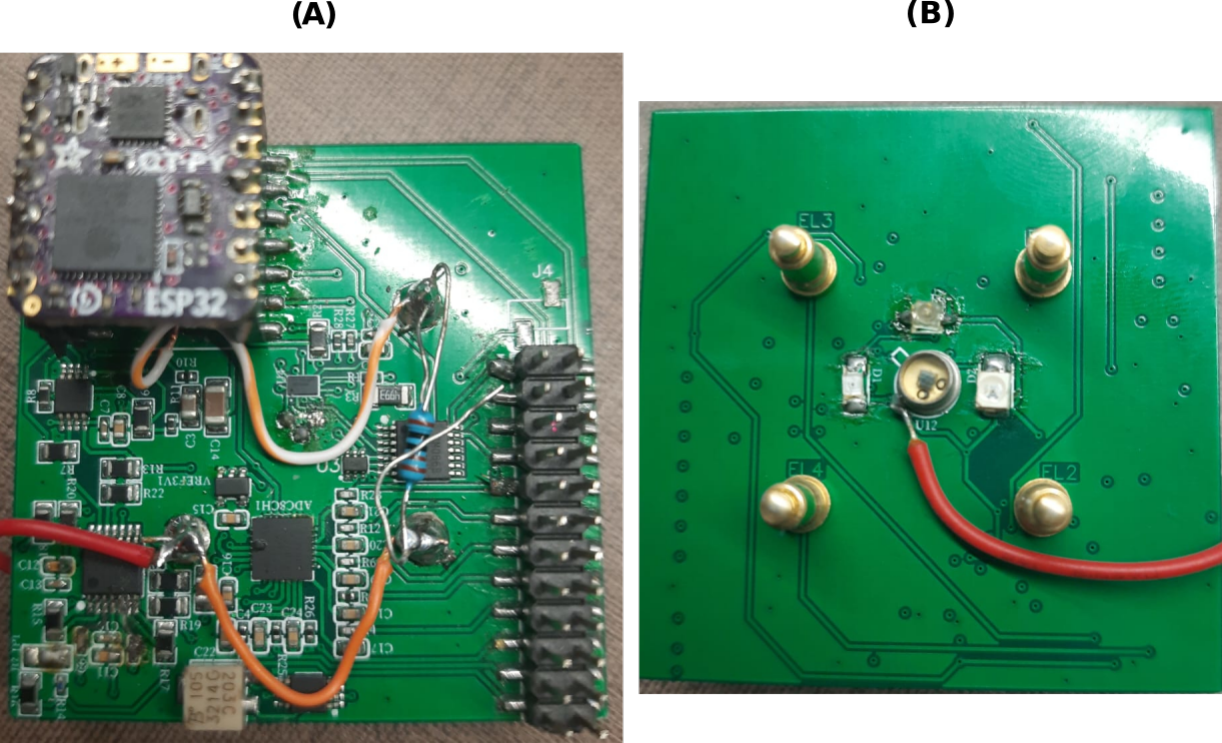
Shrink prototype (A) top view and (B) bottom view.

In addition to the current source described in this study, several modifications have simplified the power supply system shown in Fig. 2. For example, the microcontroller system has been made more compact, now operating on the same supply voltage as the entire system, with a built-in Bluetooth connection. Moreover, all components were designed using the smallest available packages. In this revised configuration, the entire system is powered by a single supply voltage from a battery, leading to a reduction in device volume. The entire measurement system is now mounted on a 5 × 5 cm PCB, as seen in Fig. 11, where (A) shows the top view of the system with its components and (B) shows the bottom view, including the bioimpedance electrodes, LEDs, and photodiode.

## Final considerations

The development of a low voltage battery supplied and instrumentation amplifier for electrical bioimpedance system was presented as a key contribution to reducing both the power consumption and the volume of a non-invasive blood glucose monitoring system. These and other improvements have led to a reduction in the overall system size, making it more suitable for future wearable applications.

However, to transform this project into a practical wearable device with minimal battery consumption, it must be miniaturized to a microscopic scale. Achieving this will require the development of an integrated circuit using CMOS technology. The initial progress toward this objective includes the work presented here as well as the research detailed in [26].

The device that incorporates the current source and the instrumentation amplifier is currently undergoing testing, and the results can be accessed through the EGluco project dashboard, available at [17].

Both simulation and bench test results confirm that the current source is robust and precise across temperature and component variations. Additionally, it has demonstrated the capability to efficiently handle rectangular signals up to 1 *MHz*, which is essential for processing DIBS-type signals at this frequency. The test results for the instrumentation amplifier demonstrated adequate performance in linearity and gain up to 100 kHz.
